# Understanding Work Ability in Employees with Pain and Stress-Related Ill-Health: An Explorative Network Analysis of Individual Characteristics and Psychosocial Work Environment

**DOI:** 10.1007/s10926-024-10200-3

**Published:** 2024-05-14

**Authors:** Hedvig Zetterberg, Xiang Zhao, Sofia Bergbom, Nadezhda Golovchanova, Ida Flink, Katja Boersma

**Affiliations:** 1 The Center for Health and Medical Psychology, School of Behavioral, Social and Legal Sciences, Örebro University, Örebro, Sweden; 2 Department of Women’s and Children’s Health, Uppsala University, Uppsala, Sweden; 3 Institute of Psychology, University of Klagenfurt, Klagenfurt am Wörthersee, Austria; 4 Department of Social and Psychological Studies, Karlstad University, Karlstad, Sweden

**Keywords:** Chronic pain, Stress symptoms, Work ability, Network analysis

## Abstract

**Purpose:**

There is a wide range of individual and work environment factors that influence work ability among workers with pain and stress-related ill-health. The multiple interactions and overlap between these factors are insufficiently understood, and a network approach could mitigate limitations of previous research. This pilot study aimed to explore interactions between individual characteristics and psychosocial work environment and potential links to long-term work ability.

**Methods:**

Prospective data from a prevention project was used. Individuals (*N* = 147) with pain and/or stress-related ill-health (95% women) at public sector workplaces filled out baseline questionnaires about a collection of individual and work environment factors, which were used for constructing undirected networks. The model was run in three subsamples of workplaces. Finally, a separate model was established with work ability at 6-month follow-up as outcome variable. A shortest pathway analysis was calculated to identify mediators of work ability.

**Results:**

Symptom catastrophizing and perceived stress were the most influential factors in all network models. Symptom catastrophizing and pain-disability risk were found to mediate the relation between perceived stress and long-term work ability. Further, demand-control-support factors were interrelated, and patterns of interaction differed between different types of workplaces.

**Conclusion:**

The findings support the importance of individual factors, specifically symptom catastrophizing in an individual’s coping with pain or stress-problems and its influence on long-term work ability. Catastrophizing might play a role in stress-related disorders which should be further investigated. Individual and work environment factors interact and vary across context, which needs to be taken into consideration to prevent pain and stress-related ill-health at work.

**Supplementary Information:**

The online version contains supplementary material available at 10.1007/s10926-024-10200-3.

## Introduction

Pain and stress-related ill-health imply huge societal challenges and suffering of those affected and are among the largest contributors to long-term sick leave [1]. These conditions are maintained through a dynamic interaction among physiological, psychological, and social factors [2, 3]. Work ability is often impaired, due to both individual and work-related factors [4–6]. However, the intricate interplay between individual and work factors has not been sufficiently studied. Further understanding of pain and stress-related ill-health calls for methods exploring the interrelations among multiple factors, not the least in relation to work ability, considering its influence on work productivity and individual health.

More than one out of five adults suffer from chronic pain [7], and the prevalence of stress-related problems might be even higher [8]. These conditions often co-occur, and are associated with poor quality of life and psychiatric co-morbidities [9]. Important factors are catastrophizing and avoidance [10], which may in turn drive negative affect, activity limitations and symptom persistence. Likewise, it is well known that factors such as beliefs about the health condition, expectations on return to work, pain-related fear, and catastrophizing are determinants of pain-related sick leave [5, 11–13]. Similarly, symptom severity and expectations on return to work have shown to predict sick leave in individuals with mental health problems [4, 14]. Considering the psychosocial work environment, factors such as high job demands and lack of social support at the workplace are associated with sickness absence and decreased work ability [4–6, 15]. The Job Demand—Control—Support model claims that employees working under high strain (a combination of high work demands, low job control, and low support) have an increased risk of health problems [16].

Importantly, the mutual dependence among this variety of individual and work environment factors has not been sufficiently studied. Most evidence on the link between ill-health and work ability build on models focusing on the unique contribution of each variable separately, where intricate interplay between factors is disregarded. Individual and work factors are often treated as separate entities, when they should rather be considered part of a network of mutually reinforcing relationships [17]. Lastly, the specific context (e.g., the workplace, the community) is rarely addressed.

A network approach could mitigate the above limitations, given the ability to represent multiple interactions qualitatively and visually. By creating a network, we regard individual characteristics and psychosocial work environment in the sample as a complex system wherein variables are interacting simultaneously, forming unique patterns [18]. So far, most studies that have used network analysis in the pain field have focused on interactions between individual (psychological) variables (e.g., [19, 20]). Others have explored work environment factors as a network system [21]. However, to our knowledge, an explorative examination using network analysis of both individual and work environment factors in a population with pain and stress-problems is absent.

Our study aimed to explore interactions between individual characteristics and psychosocial work environment among individuals with pain and stress-related ill-health and their link to long-term work ability. The following specific aims were addressed:

(1) to identify the most influential factors in terms of strength centrality in the network of baseline interactions between individual and work environment factors; (2) to explore the impact of workplace context by illustrating and comparing the above interactions at three different types of workplaces; and (3) to examine the prospective link between baseline individual and work environment factors, and work ability at follow-up.

## Methods

### Study Design

This pilot study employed a two-step prospective design, first using cross-sectional baseline data on individual and work environment factors, then adding work ability as a long-term outcome from 6-months follow-up. The prospective design allowed observation of complex interactions among the factors on work ability over time.

This study used data from an ill-health prevention project, which has been reported elsewhere [22]. The original study design was a cluster randomized controlled trial, evaluating the effects of a brief psychosocial program on sick leave and health-related outcomes among employees, compared to an active control. Notably, the intervention program had no significant effects on outcomes; thus, the entire sample was used in the current study. First-line supervisors and their employees were recruited through an occupational health care service that covers public sector workplaces such as healthcare services, schools (including pre-schools), and administrative departments. The study was approved by the Regional Ethical Review Board in Uppsala, Sweden (Number 2018/479).

### Participants

Recruitment took place at information meetings at the workplace. Inclusion criteria were as follows: (1) being employed at a workplace associated to the occupational health care service, (2) self-reported pain and/or stress-related ill-health, and (3) their immediate supervisor participated in the study. Exclusion criteria were being currently on 100% sick leave, reporting an underlying non-musculoskeletal or stress-related medical condition (e.g., cancer-related pain, hyperthyroidism) affecting work ability, or suffering from a severe psychiatric disorder (e.g., psychosis, personality disorder). All participants provided written informed consent prior to study participation.

In the current study, individuals with baseline data from the original study were included, in total 147 participants. Among these, 87 provided data on the 6-months follow-up outcome work ability. Non-responders did not differ from responders regarding demographic or baseline variables.

Participants’ characteristics are displayed in Table 1. The majority of participants were women (94.6%) with a Swedish nationality (91.2%). A high proportion (89.1%) had pain problems, where 32.8% were at risk for long-term pain disability according to screening by the OMPSQ. Levels of perceived stress (*M* = 18.73, SD = 6.25) were above levels reported from a general population (normative data of the PSS in Sweden: *M* = 14.52, SD = 6.32) [23].

**Table 1:** Participants’ characteristics for all study measures, *N* = 147

	Full sample	Health care	Administration	Schools
*Demographic variables*	*N* = 147	*n* = 76	*n* = 34	*n* = 37
Age, *M *(SD)	43.32 (10.19)	44.34 (10.69)	43.71 (9.22)	40.86 (9.84)
Women, *n* (%)	139 (94.6%)	75 (98.7%)	30 (88.2%)	34 (91.9%)
Highest education, *n* (%)				
Middle or high school	29 (19.7%)	20 (26.3%)	0 (0%)	9 (24.3%)
Vocational education	43 (29.3%)	24 (31.6%)	11 (32.4%)	8 (21.6%)
University	75 (51.0%)	32 (42.1%)	23 (67.6%)	20 (54.1%)
Born in Sweden, *n* (%)	134 (91.2%)	67 (89.3%)	31 (91.2%)	36 (97.3%)
*Pain characteristics*				
Pain prevalence, *n* (%)	131 (89.1%)	65 (85.5%)	32 (95.1%)	34 (91.9%)
Pain severity 0–10, *M *(SD) (*n* = 131)	4.43 (2.33)	4.66 (2.38)	3.94 (2.53)	4.44 (2.02)
Risk for long-term pain disability^a^ *n* (%) (*n* = 134)	44 (32.8%)	27 (35.5%)	7 (20.6%)	10 (28.6%)
*Sick leave*				
Register data sick leave past 6 months^b^ (*n* = 124)				
Prevalence of a sick leave spell, *n* (%)	19 (15.3%)	9 (13.8)	3 (8.8%)	7 (18.9%)
Total net days on sick leave, *M *(SD)	5.87 (16.71)	6.08 (18.14)	3.87 (14.86)	7.13 (15.43)
Self-reported sick leave past 12 months, *n* (%)				
0 days	28 (19.0%)	15 (19.7%)	6 (17.6%)	7 (18.9%)
1–7 days	54 (36.7%)	29 (38.2%)	13 (38.2%)	12 (32.4%
8–24 days	36 (24.5%)	17 (22.4%)	8 (23.5%)	11 (29.7%)
25–99 days	26 (17.7%)	14 (18.4%)	6 (17.6%)	6 (16.2%)
100–365 days	3 (2.0%)	3 (1.3%)	1 (2.9%)	1 (2.7%)
*Study variables at baseline*^c^ *M (SD)*				
Perceived stress (PSS)^d^	18.73 (6.25)	17.45 (6.09)	20.21 (6.05)	20.06 (6.35)
Symptom catastrophizing (SCS)^e^	5.86 (3.45)	5.57 (3.57)	5.76 (3.49)	6.54 (3.12)
Work limitations (WLQ)^f^	28.76 (14.31)	26.44 (15.06)	29.82 (9.90)	32.64 (15.20)
Pain-disability risk (OMPSQ)^g^	72.20 (38.61)	73.09 (45.15)	72.55 (29.17)	70.14 (31.40)
Health (VAS-health)^h^	56.88 (23.23)	59.05 (22.87)	54.88 (21.60)	54.24 (25.50)
Quality of life (BBQ)^i^	61.53 (20.93)	63.47 (20.37)	54.00 (20.14)	64.11 (21.74)
Support^j^	3.46 (1.06)	3.75 (0.97)	3.04 (1.08)	3.23 (1.07)
Control^j^	2.68 (1.12)	2.69 (1.09)	3.21 (1.16)	2.19 (0.96)
Demand^j^	3.33 (0.90)	3.16 (0.85)	3.42 (1.04)	3.58 (0.80)
Communication^k^	43.11 (11.14)	44.54 (10.85)	39.67 (10.67)	43.19 (11.74)
*Outcome at the 6-months follow-up*				
Work ability (WAI)^l^, *M *(SD) (*n* = 87)	37.68 (7.27)	38.55 (7.27)	36.26 (7.88)	37.35 (6.89)

^a^Above cut-off 90 on the Orebro Musculoskeletal Pain Screening Questionnaire

^b^Data from the Swedish Social Insurance Agency

^c^Missing data = 2.7%

^d^Perceived Stress Scale 0–40

^e^Symptom Catastrophizing Scale 0–14

^f^Work Limitations Questionnaire 0–100

^g^Orebro Musculoskeletal Pain Screening Questionnaire 2–210

^h^Self-rated Health 0–100

^i^Brunnsviken Brief Quality of Life Questionnaire 0–96

^j^QPS Nordic 34 + 1–5

^k^Validating and Invalidating Response Scale 0–56

^l^Work Ability Index 7–49

### Data Collection and Measures

Data were collected at baseline and 6-months follow-up by self-rating questionnaires completed online via Örebro University’s secure survey system. Information on demographics, individual, and work environment factors was collected at baseline, and on work ability at the follow-up. Swedish versions of all measures were used. Register data on sick leave were collected from the Swedish Social Insurance Agency, which manages sickness cash benefit for sick leave spells exceeding 14 days.

#### Individual Characteristics

The Perceived Stress Scale-10 (PSS-10) [23, 24] was used to measure general symptoms of stress. In the PSS, respondents rate their perception of life events during the last month as unpredictable, uncontrollable, and overloading. A five-point scale is used, ranging from never to very often. The total score ranges from 0 to 40 with higher values representing a higher stress level. The short version (10 item) has shown good reliability and validity [23, 24]. Cronbach’s alpha in this study was 0.865.

The Symptom Catastrophizing Scale (SCS) [25] was used to measure catastrophizing. SCS was developed from the Pain Catastrophizing Scale and has been evaluated for individuals with depression, demonstrating good reliability and validity [25]. Seven items cover thoughts and feelings in relation to participants’ health or mental health condition and are rated at a three-point scale. Examples of items are “I become afraid that my condition will get worse” and “I worry all the time about whether my symptoms will end.” Total scores range from 0 to 14 with higher values indicating more catastrophizing. Cronbach’s alpha in this study was 0.876.

The Work Limitations Questionnaire -16 (WLQ) [26] was used to measure disability at work, here used in relation to pain and/or stress-problems. In the WLQ, respondents rate work limitations on time management, physical demands, mental-interpersonal demands, and output demands. An index scale 0–100 is calculated, where higher scores indicate more problems. The WLQ-16 has shown acceptable psychometric properties [26]. Cronbach’s alpha in this study was 0.878.

The Orebro Musculoskeletal Pain Screening Questionnaire (OMPSQ) [27] was used to measure pain-disability risk. The OMPSQ items cover sick leave, function in daily activities, psychological status, pain-related beliefs, and recovery expectations. A total score ranges from 2 to 210, with higher values corresponding to higher risk. The OMPSQ has shown satisfactory reliability and predictive validity for long-term pain-related disability, with a cut-off score of 90 or higher indicating high risk for disability [28]. Cronbach’s alpha in this study was 0.876.

Self-rated health was measured using a visual analog scale (VAS), in horizontal digital format anchored with 0 = worst imaginable and 100 = best imaginable. Participants rated perceived their health during the last 30 days. Visual analog scales for assessing health have been evaluated extensively [29].

The Brunnsviken Brief Quality of Life Questionnaire (BBQ) [30] was used to measure quality of life, based on satisfaction and importance of different six life domains: Leisure, View on life, Creativity, Learning, Friends and Friendship, and View on self. The total scores in BBQ range from 0 to 96, where higher score indicates better outcome. The BBQ has shown high reliability and validity [30]. Cronbach’s alpha in this study was 0.832.

#### Psychosocial Work Environment

Demand, control, and support factors in the work environment were measured by subscales from the short-version General Nordic Questionnaire for Psychological and Social Factors at Work (QPS Nordic 34 +) [31, 32]. The QPS is a reliable and valid measure addressing psychosocial work environment. Items are rated on a 1–5 scale anchored from “rarely/never" to "often/always." In this study, the following subcategories and belonging items were used: *Quantitative demands;* Is your work rate unevenly distributed, leading to accumulation? Do you have too much to do? *Control over work pacing;* Can you decide your own work pacing? Can you decide when to take breaks yourself? *Support from supervisor;* If needed, do you receive support and assistance from your immediate supervisor? Do you receive appreciation for your work performance from your immediate supervisor?

Supportive communication at the workplace was measured using a modified 14-item version of the Validating and Invalidating Response Scale (VIRS) [33], here adjusted to the supervisor-employee relationship. The scale assesses communication in terms of validation (to express understanding and acknowledge the validity in a person’s experience) and invalidation (the opposite) and has previously been used to assess supportive communication among, for example, physicians [34]. A total score ranges from 0 to 56. Cronbach’s alpha in this study was 0.952.

#### Work Ability Outcome

The Work Ability Index (WAI) [35] was used to measure work ability. The WAI is a well-used measure, which has proved predictive validity for future sickness absence [36]. The WAI includes self-rated work ability in relation to demands of the work, the individual’s current health status, and mental resources, and has acceptable validity and reliability [37]. The WAI score can be categorized as poor (7–27 points), moderate (28–36 points), good (37–43 points), and excellent (44–49 points) work ability. Cronbach’s alpha in this study was 0.829.

### Analysis

Network analysis was used to address our research questions. In the network terminology, variables are represented as nodes, and edges (lines) represent their correlational relationships. Edge thickness (weighted) corresponds to strength of relationship between nodes. Data were managed using IBM SPSS 28.0 and analyses were calculated in R 4.1.2. Data distribution was first screened and severe nonnormal distributions were identified on several variables. Following the method by Liu et al. [38], a semiparametric Gaussian copula was used to treat the non-normality via the *npn* command in R-package *bootnet* [17]. Bivariate correlations between the included study variables were < 0.7. Missing data among baseline variables were 2.7%. Pairwise deletion was used to treat missing data.

As we are agnostic about the directions of associations among the variables within a network model, undirected networks were constructed, with partial correlations using the Gaussian graphical model being estimated. Specifically, we used the least absolute shrinkage and selection operator (LASSO) to produce a parsimonious network and the Extended Bayesian Information Criterion (EBIC) for optimal regularization parameter. Following the estimation, networks were plotted along with centrality metrics. Given the recent debates about interpretability of centrality metrics from psychological networks [39], we only chose strength centrality, as it means the direct influence on other network nodes. The strength centrality is the sum of the absolute edge-weights a node has in direct connections; larger strength values mean stronger and direct impact on a network. These steps were implemented with R-package *bootnet* [17] and *qgraph* [40].

To address the first research question, we estimated a network including all baseline variables. The strength centrality metrics were generated consequently, showing the more influential nodes. To address the second research question, we ran the above model in three separate subsamples, corresponding to the workplaces: health care, administrative workplaces, and schools. Permutation-based analyses (both overall and edge-wise comparisons) were conducted to investigate differences between networks from the three subsamples [41]. Notably, the EBIC tuning parameter (γ) for the subsample networks was set to 0 as the default value (*γ* = 0.5) failed to produce edges in two subsamples. This is not uncommon as 0.5 can be too conservative in practice [42]. Furthermore, we also employed the bootstrapping method (500 boot samples) to assess the edge accuracy. Most strong edges identified in the baseline network were replicable in bootstrapping tests (see Supplementary File). However, networks from subsamples administrative and school workplaces showed more instability, probably reflecting the limited sample size.

To answer the third research question, we established a separate model by including the outcome variable work ability (WAI). Apart from examining the typology of this network and the strength centrality metrics, we also utilized an algorithm by Dijkstra [43] to identify the shortest pathway from a starting node to the follow-up outcome variable (work ability). Choosing starting node was based on results from the first step (i.e., the baseline network), that is, nodes that turned out as most central according to strength metrics, as well representing measures of symptoms or health-limitations (e.g., perceived stress, health). This shortest pathway has been applied in previous empirical studies [20], which selects the route between two nodes by considering the mediation role of other nodes. In contrast to multiple regression that only shows the coefficient of predictors, the shortest pathway algorithm also visually highlights the strong mediator(s) between a pair of nodes. This step was calculated via *pathways* command in R-package *qgraph* [40].

## Results

### Interactions Between Individual Characteristics and Psychosocial Work Environment

**Fig. 1:**
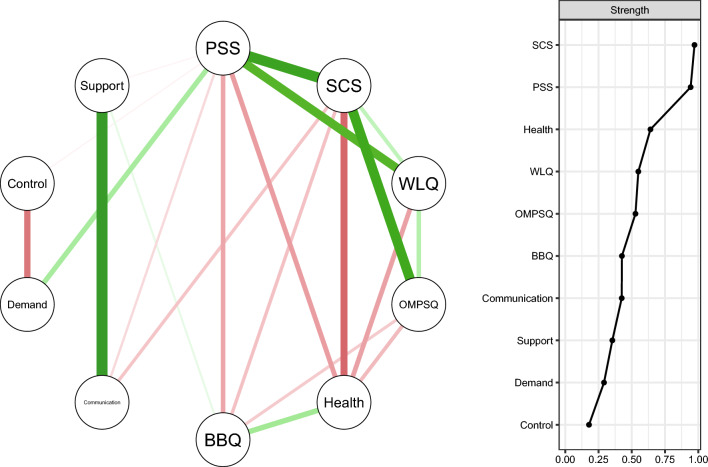
Network based on individual characteristics and psychosocial work environment factors and strength centrality plot, *n* = 147. *Note* Thicker edges indicate stronger partial correlations. Green and red edges reflect positive and negative associations, respectively. *PSS* Perceived Stress Scale, *SCS* Symptom Catastrophizing Scale, *WLQ* Work Limitation Questionnaire, *OMPSQ* Orebro Musculoskeletal Pain Questionnaire, *Health* VAS-health, *BBQ* Brunnsviken Brief Quality of life scale

First, we estimated a network based on the individual and work environment factors measured at baseline. As shown in Fig. 1, this baseline network revealed complex interactions among individual and work environment nodes. A few noticeable edges were observed between individual characteristics nodes (e.g., perceived stress (PSS)—symptom catastrophizing (SCS) as well as work environment nodes (e.g., support—communication).

Judging from the strength metrics, symptom catastrophizing (SCS) and perceived stress (PSS) showed dominant roles in the network. These influential nodes had strong interactions within other individual characteristics nodes. Notably, perceived stress was the node mostly connected with the work environment nodes. In addition, multiple connections could be observed between individual characteristics nodes, linking, for example, perceived stress and symptom catastrophizing with perceived health, work limitations (WLQ), and quality of life (BBQ). As opposed to individual characteristics nodes, work environment nodes showed less and weaker connections with other nodes. Consequently, their strength metrics were relatively lower.

### Patterns of Interactions in Three Types of Workplace Contexts

**Fig. 2:**
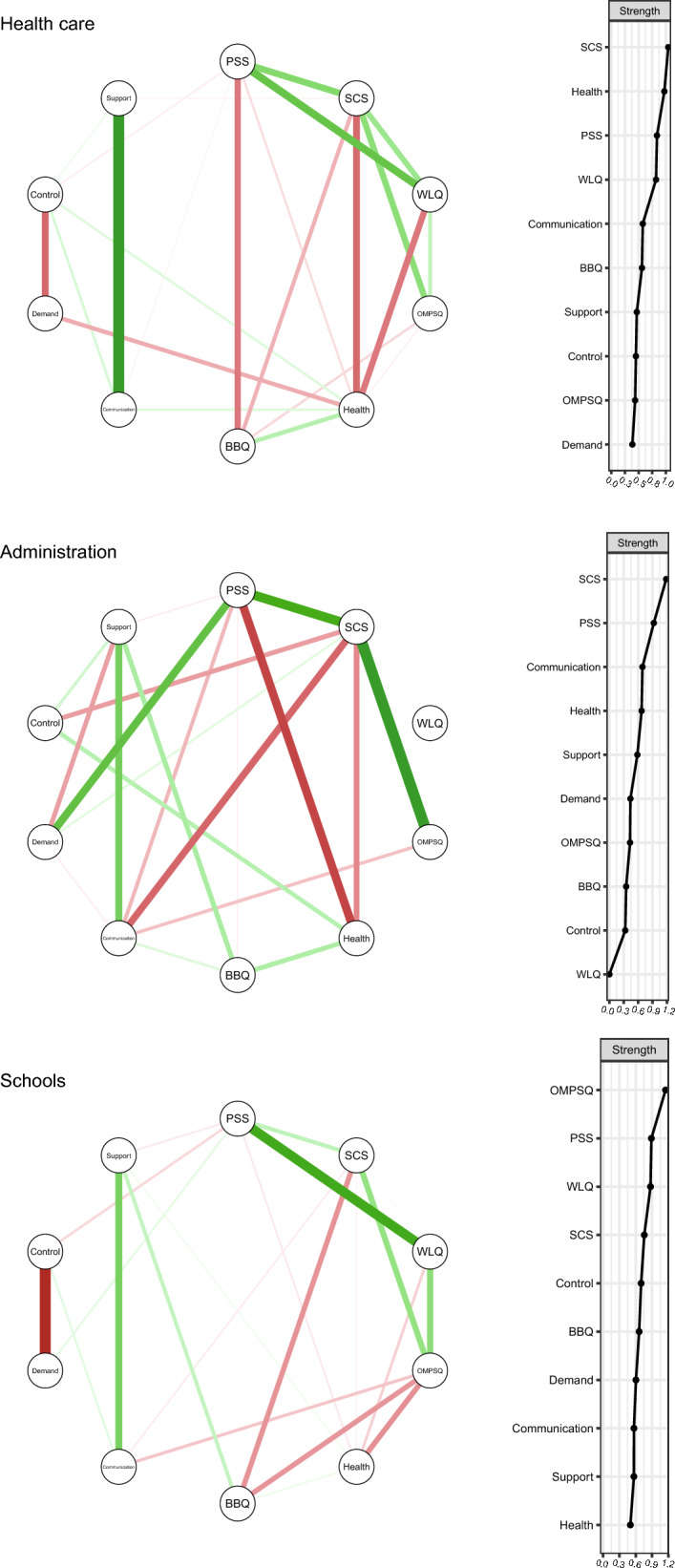
Networks based on individual characteristics and psychosocial work environment factors from three subsamples of types of workplaces, and strength centrality plots. Health care (*n* = 76), administrative (*n* = 34), schools (*n* = 37). *Note* Thicker edges indicate stronger partial correlations. Green and red edges reflect positive and negative associations, respectively. *PSS* Perceived Stress Scale, *SCS* Symptom Catastrophizing Scale, *WLQ* Work Limitation Questionnaire, *OMPSQ* Orebro Musculoskeletal Pain Questionnaire, *Health* VAS-health, *BBQ* Brunnsviken Brief Quality of life scale

Next, we constructed baseline networks for each type of workplace separately (summarized in Fig. 2). Overall, differences in the patterns of interactions could be observed in these network models. Followingly, differences in most influential nodes can be observed from the strength metrics among the three models, reflecting a variation in degree of interaction between factors in the subsamples. For example, stronger interactions between work environment nodes and individual characteristics nodes can be visually observed in the administrative workers network, with work environment nodes ranked higher in strength metrics. Symptom catastrophizing (SCS) was the most influential node in the health care workers and administrative workers’ network, and pain-disability risk (OMPSQ) was most influential in the school workers’ network.

Using permutation-based tests, we found a significant difference in structure invariance between networks of school and administrative subsamples (*M* = 0.54, *p* = 0.014). Given this result, edge-wise comparisons were calculated finding that, compared with administration’s network, the school’s network possessed stronger connections on three pairs: perceived stress (PSS)—work limitations (WLQ) (*p* = 0.001), symptom catastrophizing (SCS)—quality of life (BBQ) (*p* = 0.017), and demand—control (*p* = 0.002). Visually speaking, these edges were present in the school’s network but absent in the administration’s network (Fig. 2).

### Shortest Pathway to Work Ability at 6-Months Follow-Up

**Fig. 3:**
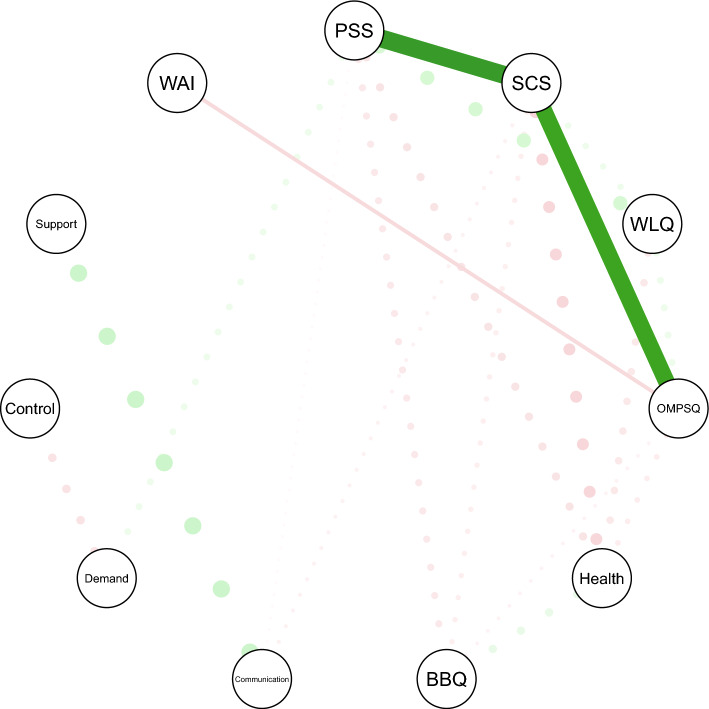
Network of individual characteristics and psychosocial work environment factors with work ability from 6-months follow-up: shortest pathways from perceived stress (PSS) to work ability (WAI), *n* = 100. *Note* Thicker edges and dots indicate stronger partial correlations. Edges indicate shortest pathway. Green and red colors reflect positive and negative associations, respectively. *WAI* Work Ability Index, *PSS* Perceived Stress Scale, *SCS* Symptom Catastrophizing Scale, *WLQ* Work Limitation Questionnaire, *OMPSQ* Orebro Musculoskeletal Pain Questionnaire, *Health* VAS-health, *BBQ* Brunnsviken Brief Quality of life scale

Work ability (WAI) from 6-months follow-up was subsequently included in the network of individual and work environment factors from baseline. As displayed in Fig. 3, work ability (WAI) showed a direct connection only to pain-disability risk (OMPSQ). Consistent with the baseline network among all participants, symptom catastrophizing (SCS) and perceived stress (PSS) had the largest strength centrality metrics (Supplementary file).

Following Dijkstra’s algorithm [43], the shortest pathways between the key node perceived stress (PSS) and the destination variable work ability (WAI) were established (Fig. 3). When all other nodes’ influences were considered in the network, the transmission from perceived stress (PSS) to follow-up work ability (WAI) was mediated by symptom catastrophizing (SCS) and pain-disability risk (OMPSQ).

## Discussion

The findings illustrate a complex pattern of multiple interactions, when exploring individual characteristics and psychosocial work environment factors and their role in work ability. The results indicated differences in patterns of interaction in subsamples from different workplace contexts. Furthermore, individual characteristics, specifically symptom catastrophizing and perceived stress, were the most influential nodes in all network models. Symptom catastrophizing and pain-disability risk were found to mediate long-term work ability.

In network analysis, the most influential nodes within networks are assumed to have the strongest direct impact on the rest of the network. Accordingly, changes in these most influential factors have the strongest potential to affect other factors in the network and overall network characteristics [17]. Based on our findings, symptom catastrophizing and perceived stress may be regarded as important targets for prevention and treatment interventions in individuals with pain and stress-related ill-health. As demonstrated by the network connectivity in our study, improvements in symptom catastrophizing and perceived stress are likely to covary with improvement in several other individual factors, such as work limitations, health, and quality of life, with which these two influential factors were strongly connected. Notably, these two factors were also reciprocally related to each other.

The major impact of catastrophizing is well in line with the pain literature, where both empirical and theoretical studies support an association with pain chronicity and disability [44, 45]. Yet, in the current study, catastrophizing is explored as a broader construct, referring to the tendency to catastrophize about different symptoms, beyond pain. Our network findings, pointing at symptom catastrophizing as a key factor, add to previous network analyses of patients with persistent pain [20]. In our study, the findings are extended to a more heterogeneous population of individuals with pain and/or stress-related ill-health.

Our findings support a strong direct link between catastrophizing and perceived stress. This relationship has previously not been well explored. Catastrophizing can be conceptualized as an emotion regulation strategy, more specifically as maladaptive problem-solving, with the function to downregulate negative affect [46], hence of relevance for both pain and mental health problems [10]. Building on this, catastrophizing (including negative repetitive thinking and worry) may influence individual’s emotional response to and appraisal of stressful events at work and in daily life. Indeed, this is in line with Lazarus transactional stress-model which emphasizes the cognitive interpretation of stressful events [47]. So far, studies on the role of emotion regulation as a mechanism in coping with work stress-problems are scarce, but regulatory strategies such as catastrophizing have been reported to be associated with burn-out among employees [48, 49].

Moreover, our findings suggest that the impact from perceived stress on work ability is mediated by catastrophizing. This is in line with the suggestion that catastrophizing can drive subsequent activity limitations in a vicious circle of negative affect and avoidance [44, 45], which in the long run may affect work ability. Taken together, our findings bring a perspective on the potential impact from catastrophizing on stress-related ill-health. This adds to the request from Fisker et al. [14], who highlighted a need for new high-quality studies on predictors for work disability, specifically for stress-related conditions. The findings in this study also strengthen the support for shared psychological mechanisms in coping with different kinds of symptoms (e.g., pain, anxiety, stressful events) [10, 50].

The other mediator of long-term work ability was pain-disability risk, measured by the OMPSQ. In addition, work ability was only directly related with the OMPSQ in the network model. Indeed, the OMPSQ is a pain screening measurement based on a composite of psychosocial risk factors, aiming to identify individuals at risk and to inform treatment. It is also known that both the OMPSQ and the WAI have shown predictive validity for long-term disability and sick leave [28, 36, 51, 52]. Poor to moderate work ability (score 7–36) demonstrates increased risk of sick leave [52], and a WAI score of ≤ 37 has been suggested to indicate need of rehabilitation among workers [53]. In this study, the average WAI score was just above 37, reflecting a subclinical sample, however with a number of individuals presenting increased needs and risk of long-term problems.

Despite the influence of some specific individual characteristics, the findings illuminate the interrelatedness with the work environment factors, informed by the demand-control-support model [16]. It could be noted that both perceived stress and symptom catastrophizing were connected, however weakly, to the work environment factors indicating that changes in psychosocial work environment could affect perceived stress. The relative larger impact from individual factors than from work environment factors in the overall network and on long-term work ability in this study aligns with previous research using regression models to identify the most influential predictors of sick leave [14, 54]. The relative small impact from work environment might also be an explanation to why preventive interventions targeting work factors lack a consistent demonstration of effect on employee ill-health [22, 55]. Still, work environment factors are indeed associated with employee outcomes [5, 15], indicating its importance, yet a broader scope, embracing both individual and work factors is needed.

Importantly, the explorative analyses indicated different network patterns at the different types of workplaces, with a variability in centrality rank among the individual and work environment factors. These preliminary findings indicate that there is a variation in degree of interrelatedness between individual factors and work environment factors, and that psychosocial work environment might be of larger importance at some workplaces. Further research is needed to assess whether this finding aligns with methods to screen for and target occupational risk factors [56]. It should be noted that physical work characteristics, known to be of importance of pain disability [5], are not addressed in this study, and adding these would further inform the network patterns.

While individual and work factors are often described as separate entities, the network findings display their connectivity, which varied across contexts. There could also be mutual relationships, for example psychological factors affecting an individual’s perception of work [56], and work factors affecting which problem-solving strategies an individual use when faced with pain and stress-problems. Kirkegaard and Brinkmann [57] argue that the appraisal and coping with work stressors are more socially embedded than as described in the transactional stress-model by Lazarus, pointing at the integration of workplace and individual perspectives.

Based on our findings, individual’s health problems and characteristics should be addressed while simultaneously taking the workplace and the work environment in consideration. This multidimensional approach is reflected in evidence-based treatments [2, 56]. However, practice lags behind recommendations and there is often a struggle to implement the multidimensional approach (e.g., involving the workplace in rehabilitation, or addressing an individual’s pain fear-beliefs in addition to physical limitations) [56]. As indicated by our findings, interrelatedness is formed differently among individuals. This can point toward idiographic approaches, adding to the knowledge derived from population-inference assumptions. Indeed, there is a promising direction with examples of idiographic networks to inform targets of treatment [58, 59]. Potentially, the level of analysis should correspond to the level of intervention, for example individual treatment or system-level prevention.

## Strengths and limitations

It should be noted that the sample predominantly included women and the results should be interpreted for women mainly. Prevalence of pain and stress-related ill-health is higher among women [2, 9, 23]; however, different patterns may exist between genders [9]. Given these potential gender differences, the connectivity among the study variables in a male sample could present different patterns, as could idiographic networks. Further, the types of workplaces in the sample did not include for instance industrial work or heavy construction work. Thus, caution needs to be taken in generalizing the results of the current study to other populations of employees as well as transferring them to an individual level.

For the subgroups, the characteristics and the network patterns should be seen as preliminary given the limited sample size. In addition, edge instability was shown in bootstrap tests for the subgroups. Other limitations are lack of clinical pain and stress diagnoses which would strengthen transferability to clinical samples, and a low response rate at 6-months follow-up. It would further have strengthened the conclusions drawn to include register data on sick leave in the analyses (sick leave at follow-up have been published elsewhere [22]). However, these data had limitations both that does not include short-term sick leave and it might have been affected by the covid pandemic.

Main strengths of this study included a prospective design and novel analytic approach. Network analysis could add to previous knowledge by its computations and illustrations of multiple bidirectional interactions between variables, and could be used to generate new hypotheses [17]. As a limitation, the small sample sizes in the network analysis calculated on subsamples of employees should be noted.

## Conclusions

The findings from this explorative study among predominantly women with pain and/or stress-related ill-health support the importance of symptom catastrophizing in an individual’s coping with pain or stress-problems and its potential role for long-term work ability. In addition, the strong interaction between perceived stress and symptom catastrophizing indicates that catastrophizing might play a role in stress-related disorders, which should be further investigated. While individual factors turned out as most central, the network models also displayed the connectivity with work environment factors, and preliminary findings that patterns could vary across contexts. This indicates that population-inference must be cautions and that an idiographic and workplace perspective need to be included for a multidimensional approach on work ability among employees with pain and stress-related ill-health.

## Supplementary Information

Below is the link to the electronic supplementary material.

Supplementary file1 (DOCX 578 kb)

## Data Availability

The data presented in this study are available on request.
