# Supplementary material for: Understanding Work Ability in Employees with Pain and Stress-Related Ill-Health: An Explorative Network Analysis of Individual Characteristics and Psychosocial Work Environment
Source: J Occup Rehabil. 2024 May 14;35(2):333–44. doi: 10.1007/s10926-024-10200-3 (PMC12089247; doi:10.1007/s10926-024-10200-3)
Supplement: Supplementary file 1 — Supplementary file1 (DOCX 578 kb) [file 10926_2024_10200_MOESM1_ESM.docx]

Supplementary materials


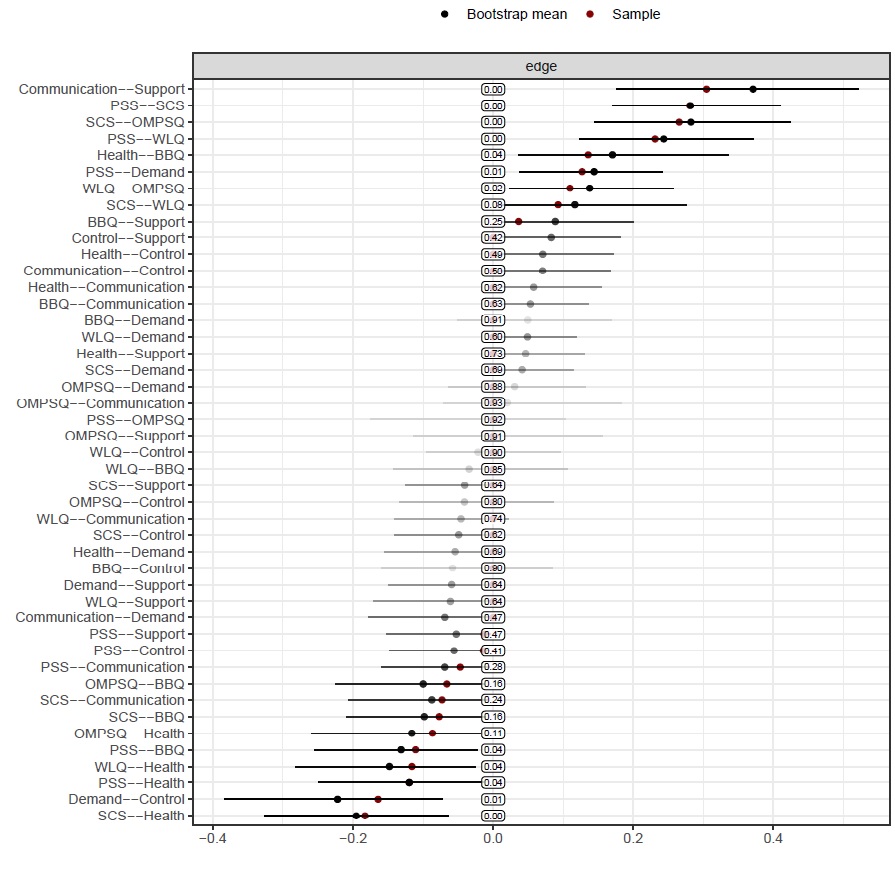


Figure S1. Results from bootstrapping analysis (500 boot samples) to assess the edge accuracy.


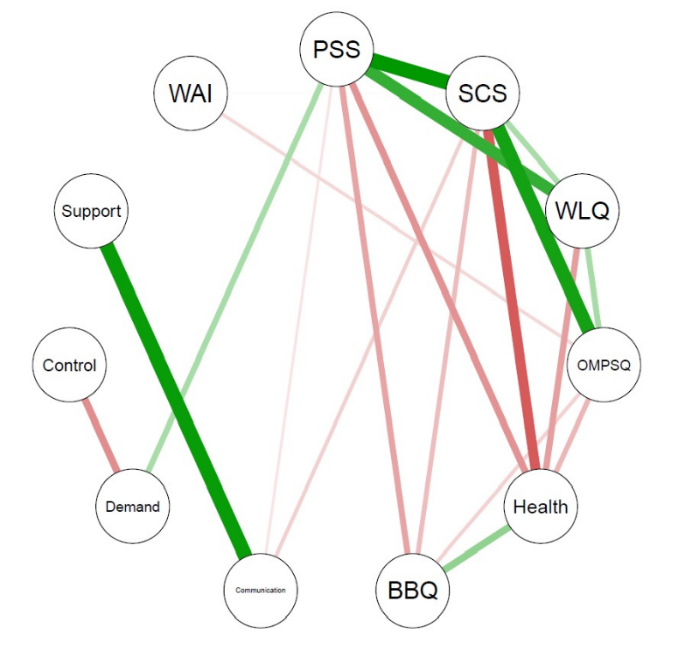

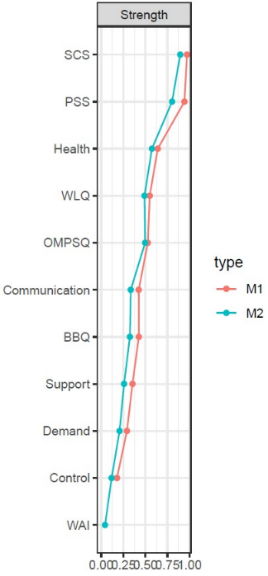


Figure S2. Network based on individual characteristics and psychosocial work environment factors with work ability (WAI) from 6-months follow-up, and strength centrality plot, n=100.

Note. Thicker edges indicate stronger partial correlations. Green and red edges reflect positive and negative associations, respectively. WAI=Work Ability Index, PSS=Perceived Stress Scale, SCS=Symptom Catastrophizing Scale, WLQ=Work Limitation Questionnaire, OMPSQ=Orebro Musculoskeletal Pain Questionnaire, Health=VAS-health, BBQ=Brunnsviken Brief Quality of life scale. In the centrality plot M1 represent baseline network only and M2 network with WAI added (Figure S2).
